# Delayed colonization of *Bifidobacterium* spp. and low prevalence of *B. infantis* among infants of Asian ancestry born in Singapore: insights from the GUSTO cohort study

**DOI:** 10.3389/fped.2024.1421051

**Published:** 2024-06-10

**Authors:** Jia Xu, Rebbeca M. Duar, Baoling Quah, Min Gong, Felicia Tin, Penny Chan, Choon Kiat Sim, Kok Hian Tan, Yap Seng Chong, Peter D. Gluckman, Steven A. Frese, David Kyle, Neerja Karnani

**Affiliations:** ^1^ Department of Human Development, Singapore Institute for Clinical Sciences, Agency for Science (SICS), Technology and Research, Singapore (A*STAR), Singapore, Singapore; ^2^ Infinant Health, Inc., Davis, CA, United States; ^3^ Department of Clinical Data Engagement, Bioinformatics Institute (BII), Agency for Science, Technology and Research (A*STAR), Singapore, Singapore; ^4^ SingHealth Duke-NUS Institute for Patient Safety and Quality, Academic Clinical Program in Obstetrics and Gynaecology, Duke-NUS Medical School, Singapore, Singapore; ^5^ Department of Maternal Fetal Medicine, KK Women’s and Children’s Hospital, Singapore, Singapore; ^6^ Department of Obstetrics and Gynecology and Human Potential Translational Research Program, Yong Loo Lin School of Medicine, National University of Singapore, Singapore, Singapore; ^7^ Centre for SPDS Centre for Informed Futures, Liggins Institute, University of Auckland, Auckland, New Zealand; ^8^ Department of Nutrition, University of Nevada, Reno, NV, United States; ^9^ Department of Biochemistry, Yong Loo Lin School of Medicine, National University of Singapore, Singapore, Singapore

**Keywords:** *Bifidobacterium longum* subsp. *infantis*, infant, ethnicity, immigration, delivery mode, antibiotics, gut microbiome, GUSTO

## Abstract

**Background:**

The loss of ancestral microbes, or the “disappearing microbiota hypothesis” has been proposed to play a critical role in the rise of inflammatory and immune diseases in developed nations. The effect of this loss is most consequential during early-life, as initial colonizers of the newborn gut contribute significantly to the development of the immune system.

**Methods:**

In this longitudinal study (day 3, week 3, and month 3 post-birth) of infants of Asian ancestry born in Singapore, we studied how generational immigration status and common perinatal factors affect bifidobacteria and *Bifidobacterium longum* subsp. *infantis* (*B. infantis*) colonization. Cohort registry identifier: NCT01174875.

**Results:**

Our findings show that first-generation migratory status, perinatal antibiotics usage, and cesarean section birth, significantly influenced the abundance and acquisition of bifidobacteria in the infant gut. Most importantly, 95.6% of the infants surveyed in this study had undetectable *B. infantis*, an early and beneficial colonizer of infant gut due to its ability to metabolize the wide variety of human milk oligosaccharides present in breastmilk and its ability to shape the development of a healthy immune system. A comparative analysis of *B. infantis* in 12 countries by their GDP per capita showed a remarkably low prevalence of this microbe in advanced economies, especially Singapore.

**Conclusion:**

This study provides new insights into infant gut microbiota colonization, showing the impact of generational immigration on early-life gut microbiota acquisition. It also warrants the need to closely monitor the declining prevalence of beneficial microbes such as *B. infantis* in developed nations and its potential link to increasing autoimmune and allergic diseases.

## 1 Introduction

Assembly of the gut microbiota during early life plays a critical role in shaping life-long immune and metabolic health. Among the first long-term colonizing gut microbes, bifidobacteria are reported to play a significant role in priming the immune system and to be protective against susceptibility to diverse diseases later in life (1, 2). However, modern lifestyle and medical practices such as the increase in the rates of cesarean section delivery, widespread use of antibiotics, and reduced breastfeeding duration are thought to have resulted in disparate rates of infants with a bifidobacteria-dominated gut microbiota between countries. Healthy infants from developing countries are more often colonized at higher abundance with bifidobacteria compared to infants from industrialized nations (3–8), where the rates of metabolic and immune-related diseases are rising (9).

Dominance of *Bifidobacterium* spp. in the gut is driven largely by the presence of human milk oligosaccharides (HMOs), which are complex carbohydrates that constitute the third most abundant component of breastmilk after lactose and lipids (10). *Bifidobacterium longum* subsp*. infantis* is unique among the bifidobacteria as it contains a large repertoire of genes encoding glycosidases and oligosaccharide transporters required to metabolize HMOs (11, 12). Furthermore, accumulating evidence suggests that *B. infantis* benefits the infant through immunoregulation of CD4+ T cells implicated in autoimmune and allergic diseases in children (13), preventing inflammation (14, 15), and improving gut barrier function (16, 17).

The highest prevalence of infant colonization with *B. infantis* has been reported in developing nations and in populations living traditional, agrarian lifestyles where breastfeeding rates are high (18–20). On the contrary, in high-income industrialized nations with a history of interrupted or low rates of breastfeeding, *B. infantis* colonization of infants is extremely rare (6–8, 20, 21). Recent findings have now shown that an initial high carriage of *B. infantis* in the gut of the breastfed infant provides a profound, durable colonization resistance (22–24). In its absence, populations of endotoxin-producing taxa, which harbour virulence and antibiotic resistance-related genes persist despite exclusive breastfeeding (23, 25), and several studies have documented evidence of high levels of enteric inflammation within the first 60 days of life among infants lacking high levels of bifidobacteria (13, 14). Retrospective studies have shown that such an early chronic enteric inflammation is associated with a dysfunction of the immune system that presents as a blunting of vaccine responses (18, 26) and increased prevalence of autoimmune disorders such as atopic dermatitis, food allergies, asthma, and Type I Diabetes (27–29).

Singapore is a high-income developed nation with high immigration and representation from three ethnic groups, Chinese (76.9%), Malays (14.6%) and Asian Indians (6.4%). Singapore has also shown an increase in the incidence of inflammatory disorders such as atopic dermatitis and allergic rhinitis in children (30, 31), affecting quality of life and raising the financial burden for the affected families and the health care system. Given the known connection between the microbiome in early life and immune health, we evaluated factors influencing the abundance and acquisition of bifidobacteria as well as the prevalence of *B. infantis* in infants from the GUSTO (Growing Up in Singapore Towards healthy Outcomes) birth cohort in Singapore.

## 2 Materials and methods

### 2.1 Study design

Infants involved in this study were part of the GUSTO birth cohort which was designed to investigate the developmental origins of health and disease (DOHaD) (32). Briefly, the GUSTO cohort recruited 1,237 pregnant women at their 1st trimesters (aged 18 and above) from two major public maternity hospitals in Singapore from 2009 to 2010 and continues to follow-up their children until now (Clinicaltrials.gov registration no. NCT01174875). All the recruited women are homogeneous Chinese, Malay, or Indian ethnic ancestry. Ethnic approvals were obtained from the SingHealth Centralised Institutional Review Board (Reference 2009/280/D) and the National Healthcare Group Domain Specific Review Board (Reference D/09/021). All participants provided the written informed consent. Written informed consent was obtained from mothers of all infants who participated in the GUSTO study. Approval for the study was granted by the ethics boards of both KK Women's and Children's Hospital (KKH) and National University Hospital (NUH), which are the Centralized Institute Review Board (CIRB) and the Domain Specific Review Board (DSRB), respectively.

To assess the factors that could influence the early colonization of bifidobacteria and prevalence of *B. infantis* in Singaporean infants, we included infants with fecal samples available longitudinally at 3 time points (day 3, week 3 and month 3 post-birth) from 3 ethnicities (Chinese, Malay, and Indian) in the current study. For this, seventy-five infants per ethnicity per time point were selected (Supplementary Table S1). All the 75 Chinese and Malay infants and 62 Indian infants had fecal samples longitudinally at 3 time points. We randomly selected some Indian infants with fecal samples available at any 2 time points or either day 3 or Month 3 to ensure all the 3 ethnicities had the same sample size of 75 at each time point. Altogether, 675 fecal samples were used in this study. General characteristics of infants included in this study are summarized in Supplementary Table S2.

### 2.2 DNA extraction

DNA extraction was performed using the QIAmp Powerfecal Pro DNA Kit (Qiagen, Hilden, Germany) as per the manufacturer's protocol with minor modifications. Briefly, approximately 0.25 g of infant fecal sample was suspended in 800 μl of CD1 solution in a PowerBead Pro tube. Samples were vortexed and bead-beaten twice for 5 min each in the TissueLyser II (Qiagen, Hilden, Germany) at 25 Hz. After the removal of inhibitors, DNA was purified through a spin column and eluted with 75 μl of pre-warmed 37°C solution C6, following which a second elution was performed to maximize the DNA yield. DNA concentration was determined by using Quant-iT PicoGreen dsDNA Assay Kit (Life Technologies, Carlsbad, California, USA).

### 2.3 Quantification of bifidobacteria and *B. infantis* with qPCR

The quantification of Real-time q-PCR was carried out using QuantStudio 6 Flex Real-Time PCR System (Applied Biosystems, Carlsbad, USA). Primers and probes (Supplementary Table S3) targeting *Bifidobacterium* spp (33). and *B. infantis* (34) were purchased from Integrated DNA Technologies (IDT, Singapore). Standard curves were generated using genomic DNA from *B. infantis* ATCC 15697. The standard DNA was diluted in a 10-fold serial dilution from 10^6^ to 10^1^ copies/reaction. For quality control, *R*^2^ value of the standard curve was >99%, and the coefficient of variation for duplications was <5%.

Reactions for the qPCR of bifidobacteria were carried out in a final volume of 25 μl containing 1× Fast SYBR green PCR mastermix (Bio-Rad, USA), 12.5 µM of forward/reverse primer, 20 ng of template DNA. The thermocycler setting consisted of an initial activation of the polymerase at 95°C for 5 min, followed by 40 cycles of 95°C for 30 s, 62°C for 20 s and 72°C for 40 s.

Reactions for the qPCR of *B. infantis* were carried out in a final volume of 15 μl containing 1× TaqMan Fast PCR mastermix (Applied Biosystems, Carlsbad, USA), 10 μM of forward/reverse primer, 5 μM probe and 2 ng of template DNA. The thermocycler setting consisted of an initial activation of the polymerase at 95°C for 30 s, followed by 40 cycles of 95°C for 30 s and 60°C for 20 s.

### 2.4 Statistical analysis

Normality tests for the abundance of bifidobacteria were performed using the Shapiro–Wilk test. As the data were not normally distributed, non-parametric tests were applied for all statistical analyses. For comparisons across different time points, Friedman's test was utilized, followed by Dunn's test for post-hoc pairwise comparisons. In the univariate analysis assessing the effects of phenotypic variables on the early colonization of bifidobacteria, the Kruskal–Wallis test was applied for variables with more than two categories, and the Mann–Whitney test was used for binary variables. For the multivariate analysis, rank-based regression models were employed to adjust for the mutual influences of significant factors identified in the univariate analysis (35). This adjustment was performed using the “Rfit” package in R version 4.3.0. The Chi-square test was employed to explore pairwise relationships between categorical phenotypic factors. All *P*-values, particularly those from multiple comparisons, were adjusted using the Bonferroni correction method to control for type I errors.

## 3 Results

### 3.1 Factors influencing colonization of Singaporean infants by bifidobacteria

**Figure 1:**
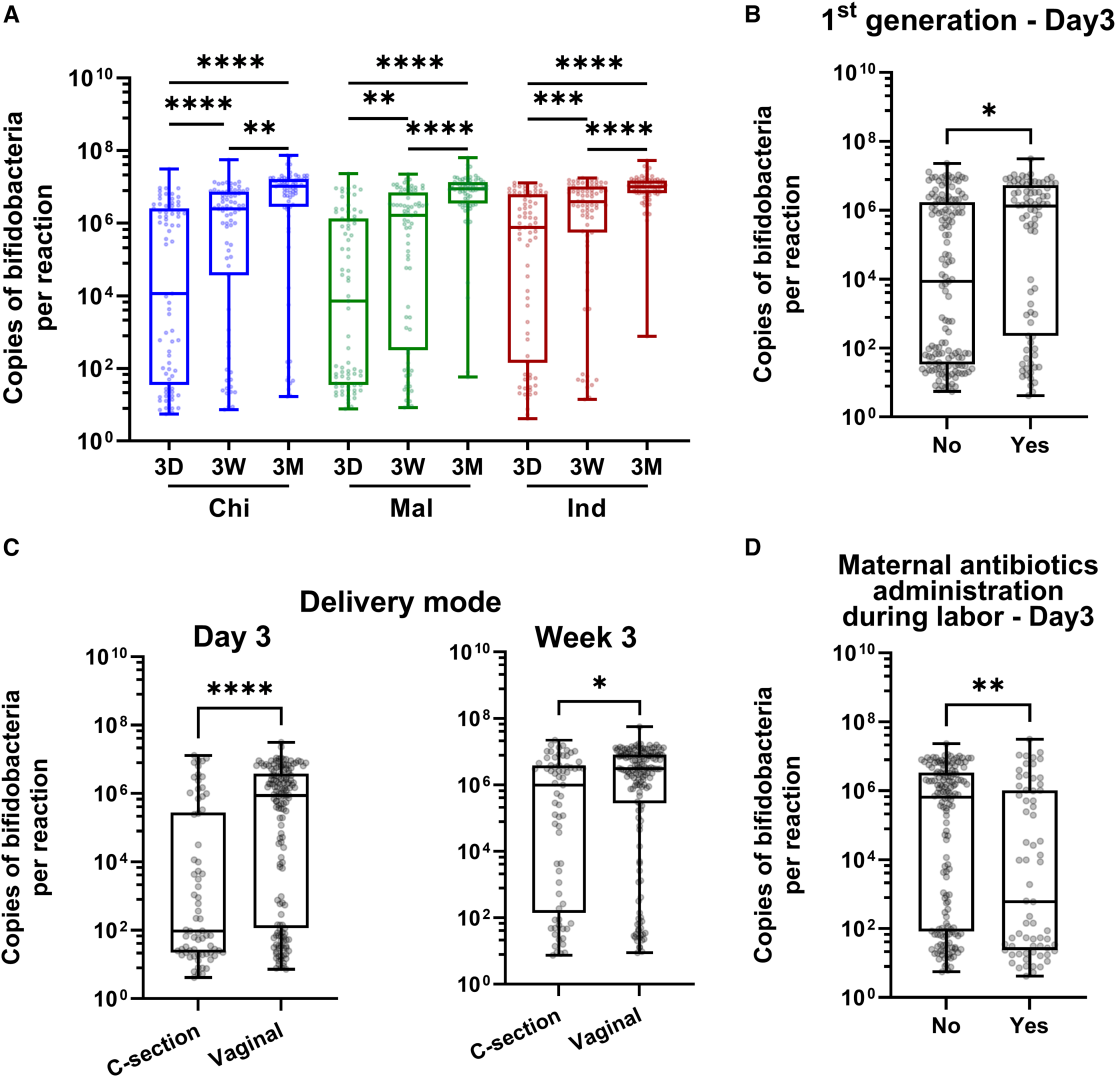
Abundance of bifidobacteria in infant gut in 3 Asian ethnic groups and its association with immigration status and clinical variables. (**A**) Longitudinal assessment of bifidobacteria abundance in infant fecal samples collected at 3 days (3D), 3 weeks (3W) and 3 months (3M) post-birth in Chinese (Chi), Malay (Mal) and Indian (Ind) ethnic groups. (**B–D**) Factors significantly associated with early colonization of bifidobacteria: First-generation status (**B**), delivery mode (**C**), and maternal antibiotics administration during labor (**D**). FDR corrected *P*-values were calculated using Dunn's post-hoc test (**A**), and rank-based regression adjusting for covariates followed by Bonferroni correction for multiple comparisons (**B–D**), where **** = *P* < 0.0001, *** = *P* < 0.001, ** = *P* < 0.01, * =*P* < 0.05 (**A**). N is 225 for 3D, 3W and 3M respectively.

To assess the factors influencing the early colonization of bifidobacteria in Singaporean infants, bifidobacteria abundance was assessed in fecal samples of all study subjects at all 3 time points (day 3, week 3 and month 3 post-birth) and across all 3 ethnicities (Chinese, Malay and Indian; *n* = 75 for each ethnicity, 675 samples in total). Bifidobacteria abundance at each time point and within each ethnic group did not show a normal distribution (Shapiro–Wilk test, *P*-value < 0.0001, Supplementary Figure S1). Over time, there was a significant increase in bifidobacteria abundance with the highest mean abundance observed at the 3-month timepoint (Figure 1A and Supplementary Table S4).

Ethnicity, first generation immigration, delivery mode, and maternal administration of antibiotics during labor significantly influenced the colonization of bifidobacteria in the univariate test (Table 1). Ethnicity and first-generation status were significantly correlated with each other (Supplementary Table S5 and Supplementary Figure S2). As ethnicity, first generation status, delivery mode, and maternal antibiotics administration during labor demonstrated significant effects in the univariate analysis, they were further examined in the multivariate analysis, adjusting for the effects of the other three factors (Table 1 and Figures 1B–D). Upon mutual adjustment, associations of first-generation status, delivery mode and maternal antibiotics administration during labor with bifidobacteria abundance at Day 3 post-birth remained significant. Additionally, the effect of caesarean-section lasted until 3 weeks post-birth (Table 1 and Figures 1B–D). Therefore, long term migratory status i.e., non-first generation, birth by caesarean-section, and maternal administration of antibiotics during labor delayed the colonization of bifidobacteria in infant gut.

**Table 1:** Univariate and multivariate analysis of factors influencing early colonization of bifidobacteria over time.

Characteristics	Univariate analysis^a^			Multivariate analysis^b^		
	Day 3	Week 3	Month 3	Day 3	Week 3	Month 3
Ethnicity	**0**.**0246**	**0**.**0354**	0.4415	NA		
Chinese vs. Malay	1	1	1	0.8300	0.7449	1
Chinese vs. Indian	0.1455	0.2315	1	0.2779	0.4314	0.6980
Malay vs. Indian	**0**.**0271**	**0**.**0355**	0.6089	0.2779	0.5002	0.6737
1st generation (Y/N)	**0**.**0022**	**0**.**0181**	0.8737	**4**.**15E-02**	0.4314	0.8402
Delivery mode (C-section vs. Vaginal)	**1**.**32E-06**	**0**.**0028**	0.0807	**1**.**39E-08**	**4**.**80E-02**	0.2380
Maternal antibiotics administration during labor (Y/N)	**0**.**0017**	0.0932	0.5925	**1**.**29E-03**	0.5002	1
Infant antibiotics administration in first 3 months (Y/N)	NA	NA	0.1898	NA		
Breastfeeding status at 3M	NA	NA	0.4596			

This table presents the *P*-values for each factor's impact on bifidobacteria colonization assessed through both univariate and multivariate analysis at Day 3, Week 3, and Month 3. *P*-values were corrected with Bonferroni method for multiple comparisons. *P*-values that are less than 0.05 after this correction are highlighted in bold.

NA, not applicable.

^a^ Kruskal–Wallis test was used for variables with more than two categories, including ethnicity and breastfeeding status at 3M. Dunn's post-hoc test was used to delineate differences between each ethnic pair. Mann–Whitney test was used for binary variables.

^b^ As ethnicity, 1st generation status, delivery mode, and maternal antibiotics administration during labor demonstrated significant effects in the univariate analysis, rank-based regression model was applied for each of these factors, adjusting for the effects of the other three factors.

### 3.2 Low prevalence of *B. infantis* in Singaporean newborns

**Figure 2:**
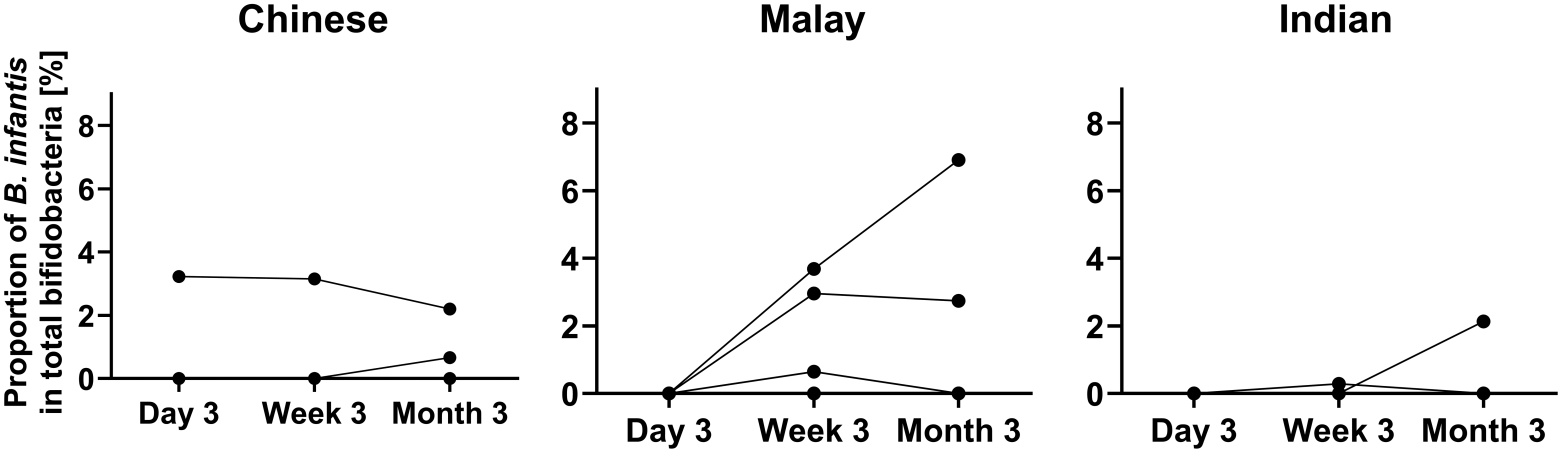
The proportion of *B. infantis* in total bifidobacteria in 10 subjects over time. *N* = 3 for Chinese, 5 for Malay, and 2 for Indian.

*B. infantis* was detected in 10/225 (4.4%) infants, and its carriage was not ethnicity dependent (Supplementary Table S6). Among the 10 subjects containing *B. infantis*, 3 were Chinese, 5 Malay, and 2 Indian. Only 1 subject (Chinese) had *B. infantis* at all three time points. The relative abundance of *B. infantis* was extremely low in the total bifidobacteria pool ranging from 0.00001 to 6.9% (Figure 2 and Supplementary Table S7). There was no consistent trend between the prevalence of *B. infantis* and factors such as antibiotics administration, mode of delivery, or duration of breastfeeding (Supplementary Table S8).

**Figure 3:**
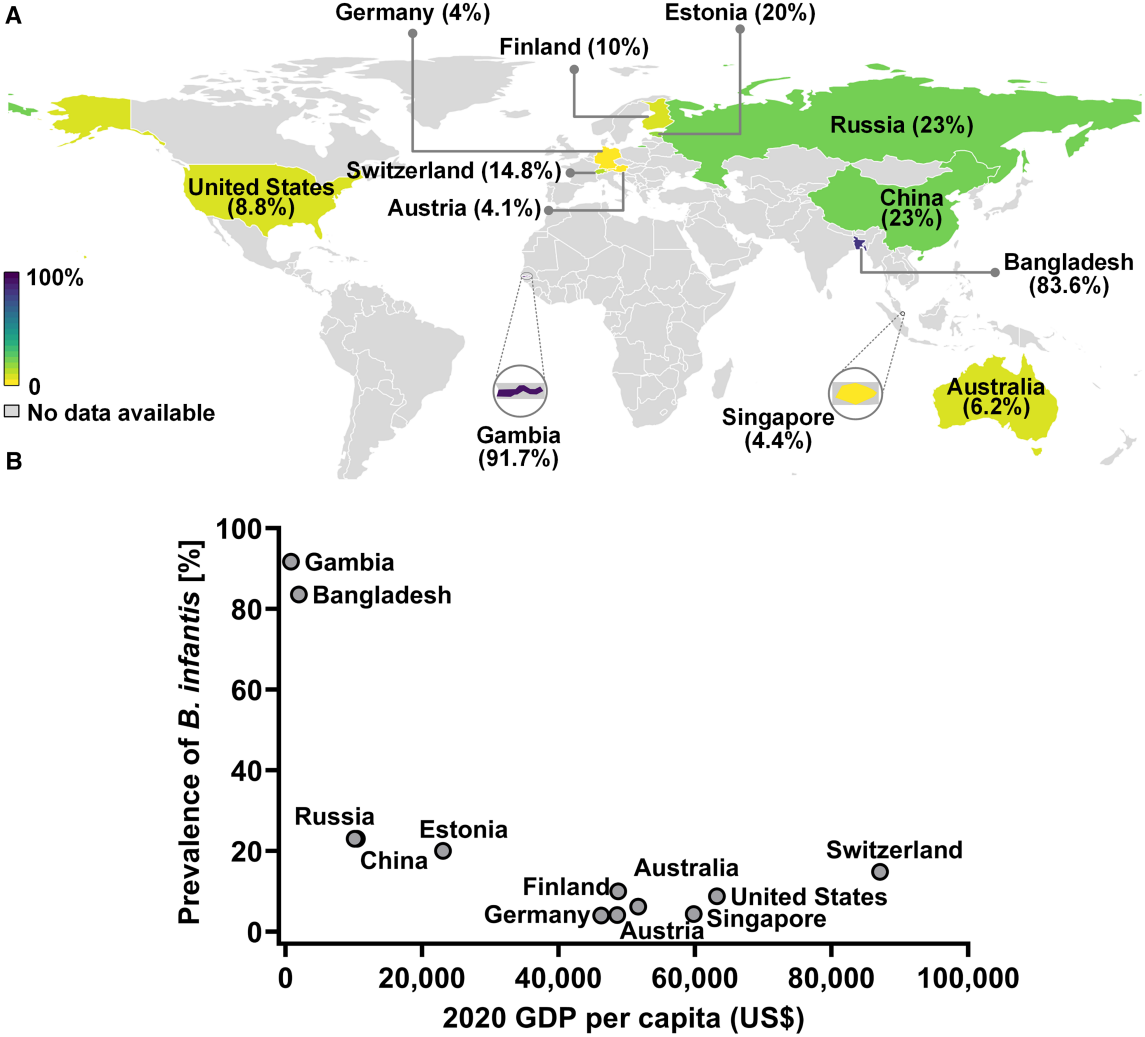
The prevalence of *B. infantis* in infant gut in developing and developed countries. (**A**) World map representation of *B. infantis* prevalence. (**B**) Negative correlation between the prevalence of *B. infantis* and the respective country's economic status as ascertained by 2020 GDP data from The World Bank (https://data.worldbank.org/indicator/NY.GDP.PCAP.CD?end=2020&name_desc=false&start=2000&view=chart).

Comparison of these results with the published datasets employing several different techniques from 11 countries identified a wide variation in the prevalence of *B. infantis* among infant populations, with trends to high (>80%), low (<25% and >5%), or very low (<5%) prevalence (Figure 3A and Supplementary Table S9). Infants from Gambia and Bangladesh are reported to have the highest prevalence of *B. infantis* (>80%). Infants from mainland China, Russia, Estonia, Switzerland, Finland, United States and Australia report the prevalence of *B. infantis* to be moderate (between 23% to 6.2%). The lowest prevalence (<5%) of *B. infantis* was reported in Germany, Austria and Singapore (Supplementary Table S9). A further analysis comparing the economic status of these countries showed a significant negative correlation between GDP per capita of the country and the prevalence of *B. infantis* (Spearman correlation, *P* = 0.0208, *R* = −0.6550; Figure 3B and Supplementary Table S9).

## 4 Discussion

In this study we identified the factors influencing colonization of bifidobacteria and the prevalence of *B. infantis* in first 3 days to 3 months of life among infants born in Singapore, a high-income, developed country. We observed use of antibiotics and cesarean section delivery delayed the colonization of bifidobacteria in the infant gut, which is consistent with previous findings (36–39). Generational immigration status was also a significant factor affecting colonization by bifidobacteria. Specifically, not being of first-generation immigration to Singapore delayed the acquisition of bifidobacteria. Similar findings have been described in adults, where immigration from developing to a developed, high-income country has a significant impact on the composition and function of microbiome (40, 41).

The reduced relative abundance of bifidobacteria among infants has been correlated to enteric inflammation and increased risk of chronic diseases as well as reduced vaccine response (13, 14, 18, 21, 26). Recent reports have suggested that loss of *Bifidobacterium* spp. in the infant born in developed countries is linked to increased incidence of allergic and autoimmune diseases (20) while carriage of *B. infantis* appears to associated with reduced risk (42). Vangay et al. tracked the gut microbiome of subjects living in China and Thailand before and after immigration to the US, and observed immigration from a non-Western country to the US decreased the gut microbial diversity, increased the ratio of *Bacteroides*/*Prevotella*, and reduced the capacity to degrade plant-derived complex carbohydrates (40). In our study, we observed a relatively higher abundance of bifidobacteria in the gut of first-generation infants born in Singapore. As the disparity in the levels of bifidobacteria in 1st generation infants was observed only at the Day 3 timepoint, our results suggest a delay in the acquisition of this microbial taxa in the non-first generation infants. Since the newborns acquire their first microbes from their environment, it's possible that modern lifestyles and practices (e.g., industrialization, urbanization, maternal diet, maternal antibiotic exposure, and other lifestyle differences) may have contributed to the delayed colonization of bifidobacteria in non-first-generation infants.

*B. infantis,* the only bifidobacteria species containing all the genes required to metabolize HMOs in human milk, was found at a very low prevalence (<5%) among Singaporean infants. This is relevant as the presence of HMO-utilization genes in the infant gut is correlated with inflammation and immune system development (13). Interestingly, the prevalence of *B. infantis* in Singapore was similar to what has been reported for other high-income developed nations. A comparative analysis of *B. infantis* in 12 countries by their GDP per capita showed a remarkably low prevalence of this microbe in advanced economies. High-income nations have also shown an increased burden of inflammatory diseases and Singapore is no exception to this trend. Incidence of allergic rhinitis in school-going children in Singapore is 40% (31), and is linked to other comorbidities including asthma, atopic dermatitis/eczema, allergic conjunctivitis and chronic sinusitis and chronic otitis media with effusion. Notably, the carriage of *B. infantis* was not ethnicity specific, indicating generic effects of urbanization and modern medical practices on infant gut microbiome.

Previous studies suggested that the colonization of *B. infantis* is associated with breastfeeding rates and antibiotic use (8). However, in our study, we did not find any consistent trends between the presence of *B. infantis* and factors such as antibiotics use, mode of delivery, or duration of breastfeeding. This could either be due to the small sample size in this study or that *B. infantis* has already diminished over generations in Singapore. Notably, there is evidence that bifidobacteria abundance (inclusive of *B. infantis*) appears to have declined from historic levels among other high-income countries (43).

This study establishes a foundational understanding of bifidobacteria colonization patterns in Singaporean infants, suggesting the significant influences of generational immigration and modern medical practices. Notably, it highlights the remarkably low presence of *B. infantis* in developed nations. We will expand our analysis to explore the diversity and abundance of different bifidobacteria species across the entire GUSTO cohort in early life, providing a more comprehensive and longitudinal view of the *Bifidobacterium* spp. that can colonize the newborn intestine. Furthermore, we intend to correlate the abundance and diversity of *Bifidobacterium* spp. with key phenotypic outcomes observed within the cohort, such as metabolic and mental health adversities, as well as allergic disorders. Understanding these relationships will further elucidate the role of early microbial colonization in the developmental origins of health and disease, paving the way for targeted interventions and proactive healthcare strategies. This approach not only deepens our understanding of microbial influences on early childhood development but also provides potential pathways for enhancing public health outcomes through precision medicine.

## 5 Conclusion

In conclusion, our study shows that immigration status and modern medical practices may influence the rates of infant colonization by bifidobacteria and sustainability of beneficial microbes such as *B. infantis* in infants born in high income developed nations, although the full health implications for infants born in high income developed nations remains unclear. Given the importance of proper microbiome assembly in early life for the development of the immune system, and the known immunomodulatory role of metabolites produced by *B. infantis* (13, 44), closer attention should be placed on the connection between the low prevalence of this important infant gut symbiont and the rise of autoimmune and allergic diseases.

## Data Availability

The clinical and demographics data supporting the conclusions of this article are available upon request from the GUSTO cohort committee (https://gustodatavault.sg/policies). The qPCR data supporting the conclusions of this article is included in the **Supplementary Material**.
