# Supplementary material for: Delayed colonization of Bifidobacterium spp. and low prevalence of B. infantis among infants of Asian ancestry born in Singapore: insights from the GUSTO cohort study
Source: Front Pediatr. 2024 Jun 10;12:1421051. doi: 10.3389/fped.2024.1421051 (PMC11194334; doi:10.3389/fped.2024.1421051)
Supplement: Supplementary file 1 [file Datasheet1.pdf]

Supplementary Material

1 Supplementary Figures and Tables

1.1 Supplementary Figures

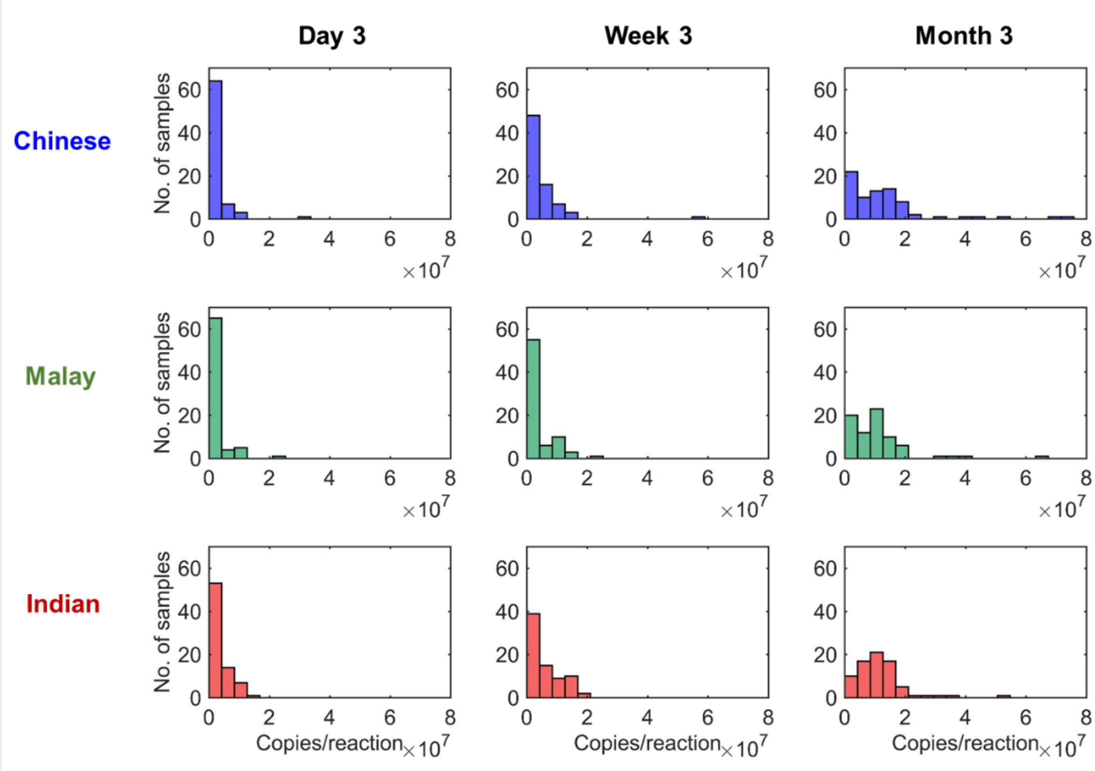

Supplementary Figure 1. Distribution of bifidobacteria abundance at 3 time points. Normality test was performed using the Shapiro-Wilk test. All P-values were <0.0001.

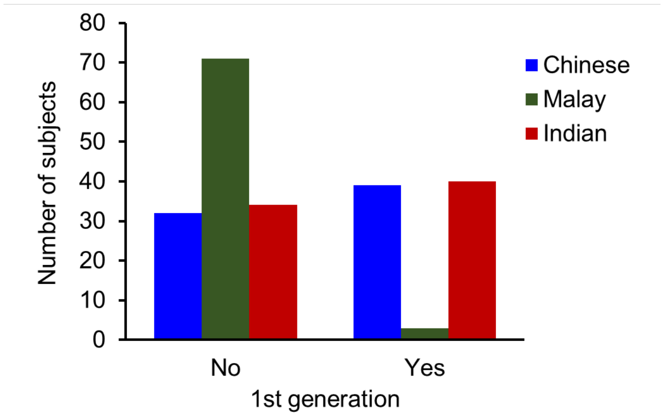

Supplementary Figure 2. Distribution of 1st generation offspring among 3 ethnicities.

1.2 Supplementary Tables

Supplementary Table 1. Number of subjects in each ethnic group

<table>
<tr><th>Ethnicity</th><th>Sample availability at all 3 time points</th><th colspan="2">Sample availability at 2 time points</th><th colspan="2">Sample availability at 1 time point</th></tr>
<tr><td>Chinese</td><td>75</td><td colspan="2" rowspan="2">NA</td><td colspan="2" rowspan="2">NA</td></tr>
<tr><td>Malay</td><td>75</td></tr>
<tr><td rowspan="3">Indian</td><td rowspan="3">62</td><td>Day3-Week3</td><td>8</td><td>Day 3</td><td>3</td></tr>
<tr><td>Day3-Month3</td><td>2</td><td colspan="2">NA</td></tr>
<tr><td>Week3-Month3</td><td>5</td><td>Month 3</td><td>6</td></tr>
</table>

Supplementary Table 2. Characteristics of subjects in this study

Characteristics	Day3	Week3	Month3
Ethnicity			
Chinese	75	75	75
Malay	75	75	75
Indian	75	75	75
1st Generation status			
Yes	82	84	83
No	137	135	136
Delivery mode			
Vaginal	155	156	155
C-section	70	69	70
Maternal antibiotics administration during labor			
Yes	67	66	67
No	158	159	158
Infant antibiotics administration in the first 3 months			
Yes	NA	NA	17
No	NA	NA	206
Breastfeeding status at Month3			
Breastmilk fed	NA	NA	23
Mixed fed	NA	NA	79
Formula milk fed	NA	NA	117

'NA' stands for 'not applicable'.

Supplementary Table 3. Primer and probe sequences for the qPCR of *Bifidobacterium* spp. and *B. infantis*.

Target	Primer/Probe	Sequence 5'-3'	Fragment length (bp)	Annealing temperature (°C)	Reference
B. longum subsp. *infantis* sialidase gene	inf_2348_F	ATACAGCAGAACCTTGGCCT	217	60	Lawley, *et al.*, 2017, PeerJ
	inf_2348_R	GCGATCACATGGACGAGAAC			
	inf_2348_P	/FAM/TTTCACGGA/ZEN/TCACCGGAC CATACG/31ABkFQ/			
Bifidobacterium spp.	Bif164F	GGGTGGTAATGCCGGATG	499	62	Satokari, *et al.*, 2001, Appl. Environ. Microbiol.
	Bif662R	CCACCGTTACACCGGGAA			

Supplementary Table 4. Abundance of bifidobacteria at Day3, Week3, and Month3

Stats		Abundance of bifidobacteria (copies per reaction)			
		**All subjects**	**Chinese**	**Malay**	**Indian**
Median (IQR)	Day3	1.94E+05 (5.38E+01-2.55E+06)	1.15E+04 (4.24E+01-2.43E+06)	7.19E+03 (3.67E+01-1.30E+06)	7.59E+05 (1.80E+02-5.99E+06)
	Week3	2.67E+06 (4.45E+04-7.61E+06)	2.46E+06 (5.17E+04-7.29E+06)	1.63E+06 (7.46E+02-5.96E+06)	3.85E+06 (5.74E+05-9.83E+06)
	Month3	9.74E+06 (5.07E+06-1.53E+07)	1.04E+07 (2.85E+06-1.65E+07)	8.81E+06 (3.51E+06-1.35E+07)	1.02E+07 (6.79E+06-1.47E+07)
Adjusted P-value*: Comparison between different time points	Day3 vs. Week3	**6.17E-11**	**6.53E-05**	**3.27E-03**	**1.08E-04**
	Day3 vs. Month3	**<1.00E-15**	**2.66E-14**	**<1.00E-15**	**<1.00E-15**
	Week3 vs. Month3	**1.10E-14**	**1.34E-03**	**3.28E-08**	**3.24E-05**

* Adjusted P-values were obtained using Dunn's test (post-hoc test after Friedman test) followed by Bonferroni correction for multiple comparisons. IQR, interquartile range.

Supplementary Table 5. Pair-wise Chi-square P-values between ethnicity, 1st generation status, delivery mode, and maternal antibiotics administration during labour

Time point	Characteristics	Ethnicity	Delivery mode	1st generation
Day3	Delivery mode	0.2201	NA	NA
	1st generation	**2.80E-12**	0.8130	NA
	Maternal antibiotics administration during labor	0.4555	0.7903	0.6952
Week3	Delivery mode	0.1839	NA	NA
	1st generation	**8.15E-13**	0.8892	NA
	Maternal antibiotics administration during labor	0.5605	0.9393	0.9834
Month3	Delivery mode	0.2201	NA	NA
	1st generation	**1.54E-12**	0.8883	NA
	Maternal antibiotics administration during labor	0.4555	0.7903	0.7646

Supplementary Table 6. Prevalence of *B. infantis*

Ethnicity	Number of subjects harboring *B. infantis*	Prevalence
Chinese	3	4.00%
Malay	5	6.67%
Indian	2	2.67%
**Total**	**10**	**4.44%**

Supplementary Table 7. Abundance trajectory of *B. infantis* and bifidobacteria in 10 subjects over time

Subject ID	Ethnicity	Copies/reaction of *B. infantis*			Copies/reaction of total bifidobacteria			*B. infantis* as a proportion of all bifidobacteria		
		Day3	Week3	Month3	Day3	Week3	Month3	Day3	Week3	Month3
S1	Chinese	8,385.1	7,944.8	277,815.8	259,757.6	251,784.8	12,608,490.3	3.22806%	3.15541%	2.20340%
S2	Chinese	0	0	78,206.1	17.2	3,842,540.2	11,736,739.1	0	0	0.66634%
S3	Chinese	0	0	1.0	7.9	4,500,652.6	16,434.5	0	0	0.00606%
S4	Malay	0	0	1.0	31.0	273,387.4	10,656,309.0	0	0	0.00001%
S5	Malay	0	0	9.4	70.8	58,299.4	3,092,991.2	0	0	0.00030%
S6	Malay	0	9.6	497,462.2	18.9	259.3	7,197,288.5	0	3.68405%	6.91180%
S7	Malay	0	317,450.8	340,102.5	79.6	10,737,997.5	12,391,111.8	0	2.95633%	2.74473%
S8	Malay	0	16.4	0	364.3	2,544.3	7,918,128.3	0	0.64553%	0
S9	Indian	0	12.6	0	701,032.2	4,346.6	6,911,117.8	0	0.29028%	0
S10	Indian	0	0	207,548.9	1,378,650.5	1,425,259.6	9,744,868.1	0	0	2.12983%

Supplementary Table 8. Phenotypes of 10 subjects harbouring *B. infantis*

Subject ID	Ethnicity	1st generation	Maternal antibiotics administration during labor	Infant antibiotics administration in first 3 months	Delivery mode	Duration of any breastfeeding	Duration of full breastfeeding
S1	Chinese	yes	no	no	C-section	<1M	<1M
S2	Chinese	no	yes	yes	Vaginal	6M~12M	6M~12M
S3	Chinese	no	yes	yes	C-section	3M~6M	<1M
S4	Malay	no	yes	no	Vaginal	NA	<1M
S5	Malay	no	yes	yes	Vaginal	<1M	<1M
S6	Malay	no	no	no	C-section	<1M	<1M
S7	Malay	no	no	no	Vaginal	6M~12M	1M~3M
S8	Malay	no	no	no	C-section	<1M	<1M
S9	Indian	yes	no	yes	Vaginal	>12M	1M~3M
S10	Indian	yes	yes	no	Vaginal	<1M	<1M

Supplementary Table 9. Summary for the prevalence of *B. infantis* in infant gut in different countries

**No.**	**Country**	**Cohort size**	**Prevalence of *B. infantis***	**Method of detection**	**2020 GDP per capita (US$)**	**Reference**
1	Gambia	24	91.7%	T-RFLP	773.00	Taft et al., 2022, Nutrients
2	Bangladesh	274	83.6%	T-RFLP	1,961.61	Taft et al., 2022, Nutrients
3	Mainland China	91	23.0% [a]	qPCR	10,434.78	Lawley et al., 2017, PeerJ
4	Russia	317	23.0%	Metagenomics	10,126.72	Vatanen et al., 2019, Nature Microbiology
5	Estonia	221	20.0%	Metagenomics	23,054.36	Vatanen et al., 2019, Nature Microbiology
6	Switzerland	227	14.8%	T-RFLP	87,100.41	Taft et al., 2022, Nutrients
7	Finland	616	10.0%	Metagenomics	48,755.36	Vatanen et al., 2019, Nature Microbiology
8	United States	430	8.8% [b]	Metagenomics	63,206.52	Casaburi et al., 2021, Scientific Reports; Tao et al., 2021, Nutrients
9	Australia	90	6.2%	qPCR	51,680.32	Lawley et al., 2017, PeerJ
10	Singapore	225	4.4%	qPCR	59,797.75	This study
11	Austria	181	4.1%	T-RFLP	48,588.66	Taft et al., 2022, Nutrients
12	Germany	198	4.0%	T-RFLP	46,252.69	Taft et al., 2022, Nutrients

[a] Calculated as the average prevalence of *B. infantis* from Chinese infants sampled over time.

[b] Calculated as the average prevalence of *B infantis* as reported by Casaburi et al., 2021 (n=227, 10% prevalence) and Tao et al., 2021 (n=203, 7.5% prevalence)
