# Supplementary figures and images for: Delayed colonization of Bifidobacterium spp. and low prevalence of B. infantis among infants of Asian ancestry born in Singapore: insights from the GUSTO cohort study
Source: Front Pediatr. 2024 Jun 10;12:1421051. doi: 10.3389/fped.2024.1421051 (PMC11194334; doi:10.3389/fped.2024.1421051)

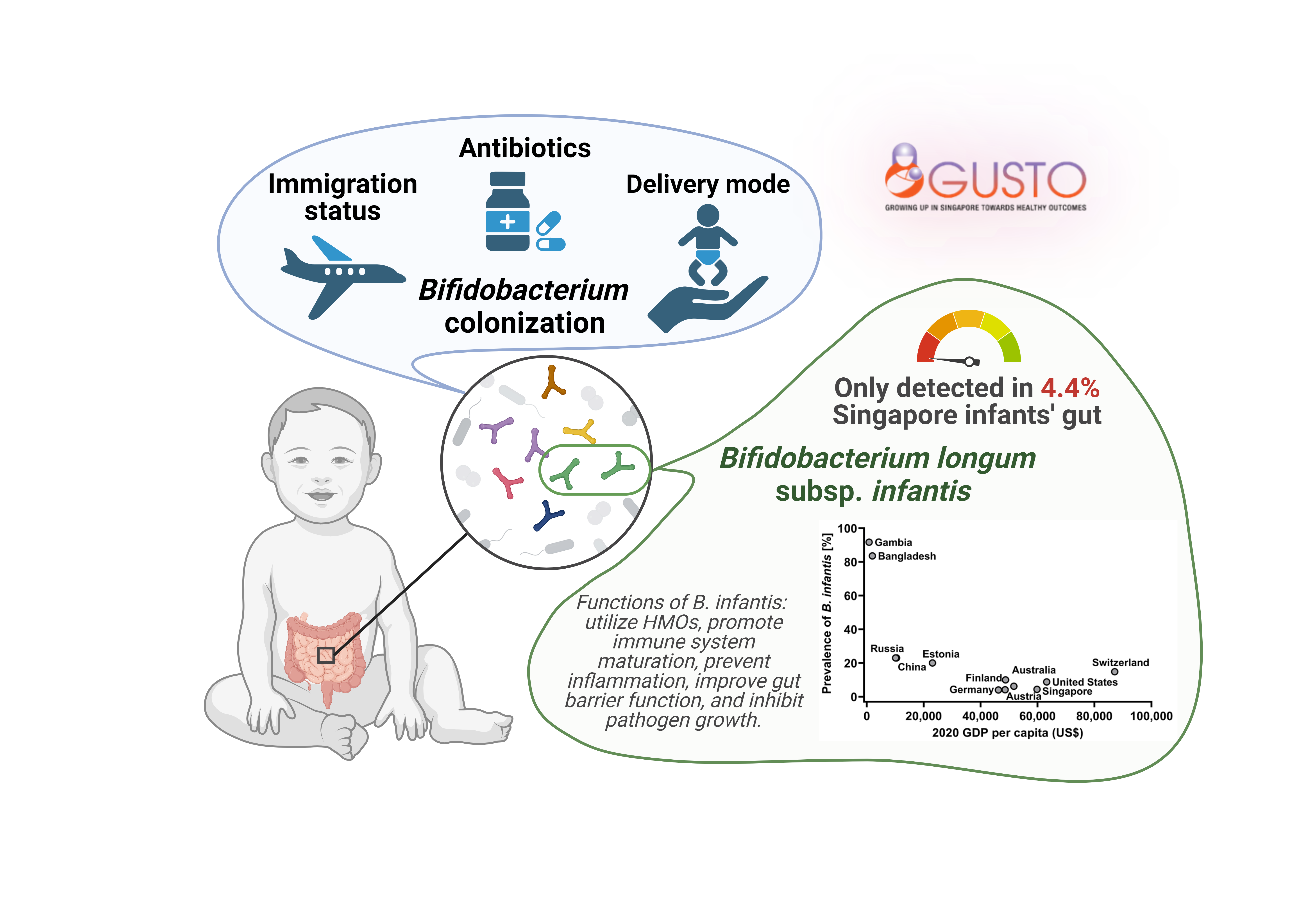

Supplement: Supplementary file 3 [file Image3.png]
